# Extent reflecting overall dietary amino acids composition adherence to the human requirement amino acids pattern is associated with the development of type 2 diabetes

**DOI:** 10.18632/aging.202777

**Published:** 2021-03-26

**Authors:** Wei Duan, Tianqi Zi, Yanhe Zhao, Ruiqi Shan, Huanyu Wu, Hu Sun, Zhen Tian, Jiemei Wang, Liyan Liu, Yuntao Zhang, Ying Li, Changhao Sun

**Affiliations:** 1 National Key Discipline, Department of Nutrition and Food Hygiene, School of Public Health, Harbin Medical University, Harbin, China; 2 Department of Epidemiology and Biostatistics, School of Public Health, Peking University Health Science Center, Beijing, China

**Keywords:** amino acids, compositions, quality index, type 2 diabetes mellitus

## Abstract

This study aimed to elucidate whether dietary amino acids (AAs) composition is associated with type 2 diabetes mellitus (T2DM) and to investigate how serum AAs profiles mediated this association. Two prospective cohorts of 1750 and 4024 adults were enrolled. Dietary AAs compositions index (AACI) was developed to reflect the overall quality of dietary AAs composition. Multivariate linear regression and logistic regression models were used to examine associations of AACI and T2DM. The AACI was associated with the incidence of T2DM with the relative risk and 95%CI from the bottom to the top tertiles being 1.00, 1.49 (0.88-2.51) and 2.27 (1.20-4.28), and 1.00, 1.58 (1.13-2.19) and 2.33 (1.56-3.47) in the two cohorts, respectively. The AACI was positively associated with serum valine, isoleucine, glutamic acid and phenylalanine, and it was negatively associated with serum glycine and histidine in both cohorts (*P*<0.01). Valine, glutamic acid and histidine consistently and partially mediated the association between the AACI and T2DM in the two cohorts, with total mediation effects of 33.4% and 54.6%, respectively. Dietary AAs composition was associated with the incidence of T2DM, meanwhile, the relationship was mediated by some degree of serum AAs. Future dietary strategies should focus on the improvement of the overall quality of dietary AAs compositions.

## INTRODUCTION

Amino acids (AAs) have been increasingly studied as playing roles in the development of insulin resistance and type 2 diabetes mellitus (T2DM) [1, 2]. However, current studies regarding the relationship between dietary AAs and T2DM have been frequently inconsistent [3–7], and it is still largely unknown whether and how serum AAs mediated the relationship between dietary AAs and T2DM.

Previous studies commonly focused on relationships between an individual dietary amino acid and T2DM [3–7], but ignored that different dietary AAs composition in the overall diet may influence the biological value of protein intake, resulting in different absorbed-, utilized- and metabolic-rates of AAs [8–11], which may contribute to these inconsistent results. Further, the varied absorbed-, utilized- and metabolic-rates of AAs in different dietary AAs composition also make it difficult to capture the link between an individual amino acid and its plasma levels. Therefore, intake of a single kind of amino acid may not be commonly reflected in its serum amino acid level, meanwhile, making it remain controversial whether the relationship between dietary AAs and T2DM was mediated by serum AAs [3, 12–14]. These key questions probably hampered the development of useful dietary guidelines of AAs in the prevention and management of T2DM.

The concept of human requirement amino acids pattern (HRAAP) may provide clues for solving these questions. It provides and emphasizes the necessity of a suitable composition of dietary essential AAs to achieve optimal amino acid metabolism, which could maintain the normal function of tissues and organs in the body [15]. However, there is still no study that has assessed whether this concept could be applied in the field of T2DM. Based on this concept, we hypothesized that to prevent T2DM, overall dietary AAs compositions should adhere to the HRAAP. The closer adherence to the HRAAP, the higher absorbed-, utilized- and metabolic-rates of AAs in the body were, which thereby maintained plasma AAs profiles at appropriate levels. Otherwise, the absorbed-, utilized- and metabolic-rates of AAs will be influenced, showing disordered plasma AAs profiles, and some dysregulated AAs may result in insulin resistance and subsequent T2DM.

To validate our hypothesis, we intended to construct dietary AAs compositions index (AACI) to reflect the extent to which overall dietary AAs compositions adhere to the HRAAP, and examined the association between AACI and future risk of T2DM in two prospective cohorts. Once the association between AACI and T2DM was confirmed, we intended to further clarify whether the association between the AACI and T2DM was mediated by serum AAs that to provide complete evidence in this issue.

## RESULTS

### Baseline characteristics of participants in the two cohorts

Participants in the HDNNCDS were older, and had higher alcohol consumption rate, calorie intake, protein intake and saturated fat intake than those in the HPHS. Fasting glucose, TC, TG, LDL-C were significantly higher in the HDNNCDS than those in the HPHS (Supplementary Table 1). The mean levels of study variables according to tertiles of AACI were presented in Table 1. In the HDNNCDS, as the AACI at baseline increased from the bottom to the top tertile, the proportion of men, smoking rate, alcohol rate, BMI gradually increased, and protein intake, fiber intake, saturated fatty acid intake, TC levels, HDL-C levels gradually decreased. In the HPHS, BMI gradually increased, and protein intake, saturated fatty acid intake, HDL-C gradually decreased (*P*<0.05 for all cases).

**Table 1:** Baseline characteristics of participants by tertiles of AACI in the HPHS and HDNNCDS.

	**HPHS**				**HDNNCDS**			***P*-value**
	**Tertile 1** **(N=584)**	**Tertile 2** **(N=582)**	**Tertile 3** **(N=584)**	***P*-value**	**Tertile 1** **(N=1341)**	**Tertile 2** **(N=1341)**	**Tertile 3** **(N=1342)**	
Age (years)	44.9 (10.7)	46.5 (10.3)	46.4 (10.3)	0.017	48.7 (9.7)	49.6 (9.4)	50.3 (9.6)	<0.001
Men [n (%)]	149 (25.5)	194 (33.3)	194 (33.2)	0.005	362 (27.0)	454 (33.9)	524 (39.0)	<0.001
BMI (kg/m^2^)	24.7 (3.5)	25.3 (3.4)	25.4 (3.5)	0.018	24.6 (3.4)	24.9 (3.5)	25.1 (3.5)	0.047
Regular exercise habits [n (%)]	327 (56.0)	331 (56.9)	333 (57.0)	0.929	642 (47.9)	617 (46.0)	621 (46.3)	0.578
Over senior middle school [n (%)]	393 (67.3)	374 (64.3)	346 (59.2)	0.015	1056 (78.7)	985 (73.5)	858 (63.9)	<0.001
Current smokers [n (%)]	72 (12.3)	92 (15.8)	94 (16.1)	0.253	181 (13.5)	198 (14.8)	243 (18.1)	<0.001
Current drinkers [n (%)]	177 (30.3)	171 (29.4)	155 (26.5)	0.333	505 (37.7)	433 (32.3)	475 (35.4)	0.014
Energy intake (kcal/day)	2237 (941)	2177 (748)	2345 (832)	0.003	2297 (801)	2311 (1000)	2530 (830)	<0.001
Protein (g/day)	75.7 (24.7)	64.5 (25.5)	63.8 (24.8)	<0.001	78.0 (50.1)	69.4 (28.6)	70.1 (26.2)	<0.001
Fiber (g/day)	13.6 (8.5)	14.3 (6.7)	14.7 (6.3)	0.061	12.4 (7.0)	14.4 (6.7)	15.3 (6.9)	<0.001
Saturated fatty acid (g/day)	18.1 (8.0)	14.7 (5.9)	12.0 (4.3)	<0.001	19.3 (10.8)	15.9 (6.9)	13.5 (5.1)	<0.001
Fasting glucose (mmol/L)	4.65 (0.68)	4.71 (0.68)	4.76 (0.73)	0.104	4.53 (0.64)	4.53 (0.72)	4.49 (0.72)	0.294
2-hour glucose (mmol/L)	5.67 (1.64)	5.69 (1.68)	5.66 (1.69)	0.892	5.74 (1.59)	5.78 (1.62)	5.88 (1.70)	0.079
HbA1c (%)	4.95 (0.51)	5.00 (0.56)	5.08 (0.61)	<0.001	5.52 (0.87)	5.52 (0.93)	5.67 (0.63)	<0.001
Fasting insulin (μU/mL)	8.31 (6.94)	8.37 (9.15)	8.49 (9.91)	0.964	8.51 (6.20)	8.75 (12.4)	8.39 (7.89)	0.727
TG (mmol/L)	1.66 (1.22)	1.75 (1.32)	1.79 (1.39)	0.568	1.62 (1.57)	1.67 (1.53)	1.79 (1.73)	0.345
TCHO (mmol/L)	4.94 (0.93)	4.86 (0.91)	4.93 (0.95)	0.157	5.20 (1.03)	5.08 (0.98)	5.09 (1.01)	0.001
HDL-C (mmol/L)	1.33 (0.32)	1.28 (0.32)	1.24 (0.32)	<0.001	1.32 (0.33)	1.26 (0.32)	1.22 (0.31)	<0.001
LDL-C (mmol/L)	2.93 (0.98)	2.88 (0.97)	2.80 (0.96)	0.062	3.01 (0.87)	2.97 (0.82)	3.02 (0.88)	0.323

Mean ± Standard Deviation was used for continuous variables.

One-way ANOVA was used for continuous variables; Chi-square test was used for categorical variables. BMI, body mass index; TG, triglyceride; TCHO, total cholesterol; HDL-C, high-density lipoprotein cholesterol; LDL-C, low-density lipoprotein cholesterol; HbA1c, glycosylated hemoglobin; HPHS, Harbin People health Study; HDNNCDS, the Harbin Cohort Study on Diet, Nutrition and Chronic Noncommunicable Disease.

### Association between AACI and incidence of T2DM

Associations between AACI and incidence of T2DM in the two cohorts were presented in Table 2. In the HPHS, compared with participants in the lowest tertile of AACI, the RRs (95% CIs) for those in the second and third, were 1.50 (95% CI 0.90–2.51) and 2.19 (1.16–4.11) (*P*_for trend_=0.015), with adjustment for demographic and nutritional covariates. When the model additionally included biochemical indices, this association become marginally significant. The RRs (95% CIs) were 1.00 (reference), 1.49 (0.88–2.51) and 2.27 (1.20–4.28) (*P*_for trend_=0.012). In the HDNNCDS, compared with participants in the lowest tertile of AACI, the RRs (95% CIs) for those in the second and third, were 1.55 (95% CI 1.12–2.15) and 2.28 (1.54–3.39) (*P*_for trend_=0.001), with adjustment for demographic and nutritional covariates. When the model additionally included HOMA-IR and blood lipid profiles, the association between the AACI and risk of T2DM remained significant. The RRs (95% CIs) were 1.00 (reference), 1.58 (1.13–2.19) and 2.33 (1.56–3.47) (*P*_for trend_=0.001). The two cohorts consistently showed that increased AACI was associated with an increased risk of T2DM. In the multivariable regression models (Table 2), the standardised regression coefficients (β) of AACI to HbA1c were 0.084 (*P*=0.003) and 0.036 (*P*=0.049) in the HPHS and HDNNCDS, respectively, after adjustment for all the above covariates. The two cohorts consistently showed positive association between AACI and HbA1c.

**Table 2:** RRs (95% CI) of the incidence of T2DM across tertiles of AACI in the two cohorts.

**AACI**	**Case/N**	**Model 1**	**Model 2**	**Model 3**	**Model 4**
HPHS [RR (95%CI)]					
<3.32	35/584	1	1	1	1
3.32-3.57	51/582	1.44(0.92-2.28)	1.34(0.84-2.13)	1.50(0.90-2.51)	1.49(0.88-2.51)
>3.57	62/584	1.82(1.18-2.82)	1.71(1.09-2.68)	2.19(1.16-4.11)	2.27(1.20-4.28)
*p* for trend		0.007	0.019	0.015	0.012
HDNNCDS (RR [95%CI])					
<3.36	104/1341	1	1	1	1
3.36-3.53	130/1341	1.36(1.02-1.82)	1.27(0.95-1.71)	1.55(1.12-2.15)	1.58(1.13-2.19)
>3.53	151/1342	1.67(1.26-2.22)	1.50(1.12-1.99)	2.28(1.54-3.39)	2.33(1.56-3.47)
*p* for trend		0.001	0.006	0.001	0.001
HbA1c [β (*P*-value)]					
HPHS	88/1750	0.083(<0.001)	0.065(0.005)	0.084(0.003)	0.084(0.003)
HDNNCDS	255/4024	0.053(<0.001)	0.044(0.006)	0.037(0.040)	0.036(0.049)

Data are RRs (95%CI) or β (*P*-value).

Model 1 was crude model;

Model 2 was further adjusted by demographic covariates including age, gender, BMI, education, alcohol consumption rate, smoking rate and regular exercise habits;

Model 3 was further adjusted by nutritional covariates including dietary energy intake, protein intake, carbohydrate intake, lipid intake, fiber, cholesterol, saturated fatty acid, monounsaturated fatty acids, polyunsaturated fatty acids and overall diet quality;

Model 4 was further adjusted by biochemical indices including total cholesterol, triglyceride, high-density lipoprotein cholesterol, low-density lipoprotein cholesterol and HOMA2-IR.

### Association of AACI with serum AAs profiles

Associations of AACI with serum AAs profiles in the two cohorts were presented in Table 3. In the HPHS, AACI was positively associated with levels of serum glutamine, valine, isoleucine, glutamic acid, phenylalanine, and it was negatively associated with levels of glycine, proline, histidine (all the *P*<0.01). In the HDNNCDS, AACI was positively associated with leucine, valine, isoleucine, serine, alanine, phenylalanine, tryptophan, and it was negatively associated with levels of glycine and histidine (all the *P*<0.01). The AACI was consistently associated with six of eighteen serum AAs including valine, isoleucine, glycine, glutamic acid, phenylalanine, histidine in the two cohorts.

**Table 3:** The associations between AACI and serum amino acids profiles.

	**HPHS**			**HDNNCDS**		
	Model 1	Model 2	Model 3	Model 1	Model 2	Model 3
Threonine	0.035	0.032	0.053	0.018	0.025	0.029
Glutamine	0.116^**^	0.116^**^	0.119^**^	0.006	0.007	0.011
Leucine	0.025	0.003	0.012	0.107^**^	0.086^**^	0.084^**^
Arginine	-0.016	-0.013	0.013	0.024	0.041^**^	0.043^**^
Valine	0.134^**^	0.125^**^	0.148^**^	0.042^**^	0.045^**^	0.042^**^
Isoleucine	0.146^**^	0.130^**^	0.147^**^	0.117^**^	0.096^**^	0.088^**^
Serine	0.033	0.042	0.051	0.011	0.038^*^	0.045^**^
Methionine	-0.013	-0.019	-0.005	0.031	0.010	0.007
Glycine	-0.074^*^	-0.070^*^	-0.080^*^	-0.069^**^	-0.055^**^	-0.050^**^
Alanine	0.009	0.007	0.012	0.079^**^	0.075^**^	0.075^**^
Lysine	0.006	-0.007	0.005	0.011	-0.003	-0.006
Glutamic acid	0.126^**^	0.120^**^	0.115^**^	0.097^**^	0.089^**^	0.083^**^
Aspartic acid	-0.009	-0.011	-0.008	0.025	0.027	0.033
Tyrosine	0.005	0.005	0.033	0.014	0.023	0.018
Phenylalanine	0.070^**^	0.090^**^	0.090^**^	0.100^**^	0.115^**^	0.116^**^
Tryptophan	-0.016	-0.021	-0.017	0.063^**^	0.074^**^	0.073^**^
Proline	-0.078^*^	-0.097^**^	-0.098^**^	0.023	0.019	0.018
Histidine	-0.092^**^	-0.142^**^	-0.117^**^	-0.067^**^	-0.122^**^	-0.109^**^

Data are standard coefficients in the multivariate regression analysis.

Model 1 no adjustment;

Model 2 was adjusted by age, gender, body mass index, alcohol, smoke, regular exercise habits, education level and family history of diabetes;

Model 3 was adjusted by all variables in model 2 and total calorie intake, dietary protein intakes, dietary fiber;

*P<0.05 for the coefficients being different from 0; **P<0.01 for the coefficients being different from 0.

### Association of serum AAs profiles with T2DM

**Figure 1:**
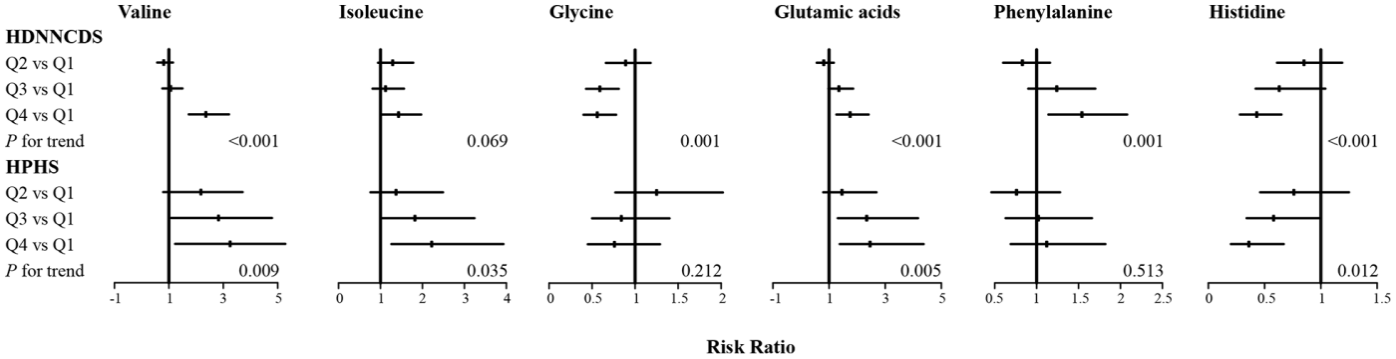
**Associations of six serum amino acids with incidence of type 2 diabetes in HPHS and HDNNCDS.** Data are RR and its 95%CI with adjustment for age, gender, BMI, education, alcohol consumption rate, smoking rate, regular exercise habits, dietary energy intake, protein intake, fiber, saturated fatty acid, overall diet quality, AACI, total cholesterol, triglyceride, high-density lipoprotein cholesterol, low-density lipoprotein cholesterol and HOMA2-IR; HPHS, Harbin People health Study; HDNNCDS, the Harbin Cohort Study on Diet, Nutrition and Chronic Noncommunicable Disease.

As six serum AAs were consistently observed to be associated with AACI in the two cohorts, the associations of these serum AAs and risk of T2DM were further analysed in the two cohorts in Figure 1 (more detailed data are given in Supplementary Table 2). In the HPHS, after adjustment for covariates, valine, isoleucine, glutamic acid and histidine were associated with T2DM, with the RRs (95%CI) from the bottom to the top quartiles being 1 (reference), 1.71 (0.79-3.71), 2.28 (1.08-4.79), 2.55 (1.23-5.28) for valine; 1 (reference), 1.37 (0.76-2.49), 1.82(1.02-3.24), 2.22 (1.26-3.93) for isoleucine; 1 (reference), 1.46 (0.79-2.69), 2.34 (1.31-4.17), 2.46 (1.38-4.37) for glutamic acid; and 1 (reference), 0.76 (0.46-1.25), 0.58 (0.34-0.99), 0.36 (0.20-0.67) for histidine. In the HDNNCDS, valine, glycine, glutamic acid, phenylalanine and histidine were associated with T2DM, with the RRs (95%CI) from the bottom to the top quartiles being 1 (reference), 0.81 (0.57-1.15), 1.07 (0.76-1.50), 2.36 (1.73-3.21) for valine; 1 (reference), 0.89 (0.66-1.18), 0.59 (0.43-0.81), 0.56 (0.40-0.78) for glycine; 1 (reference), 0.81 (0.56-1.17), 1.35 (0.98-1.86), 1.75 (1.26-2.41) for glutamic acid; 1 (reference), 0.83 (0.60-1.16), 1.24 (0.90-1.70), 1.54 (1.14-2.08) for phenylalanine and 1 (reference), 0.85 (0.61-1.19), 0.63 (0.42-1.04), 0.43 (0.28-0.65) for histidine. Valine, glutamic acid and histidine were consistently associated with T2DM in the two cohorts.

### Mediation analysis

**Figure 2:**
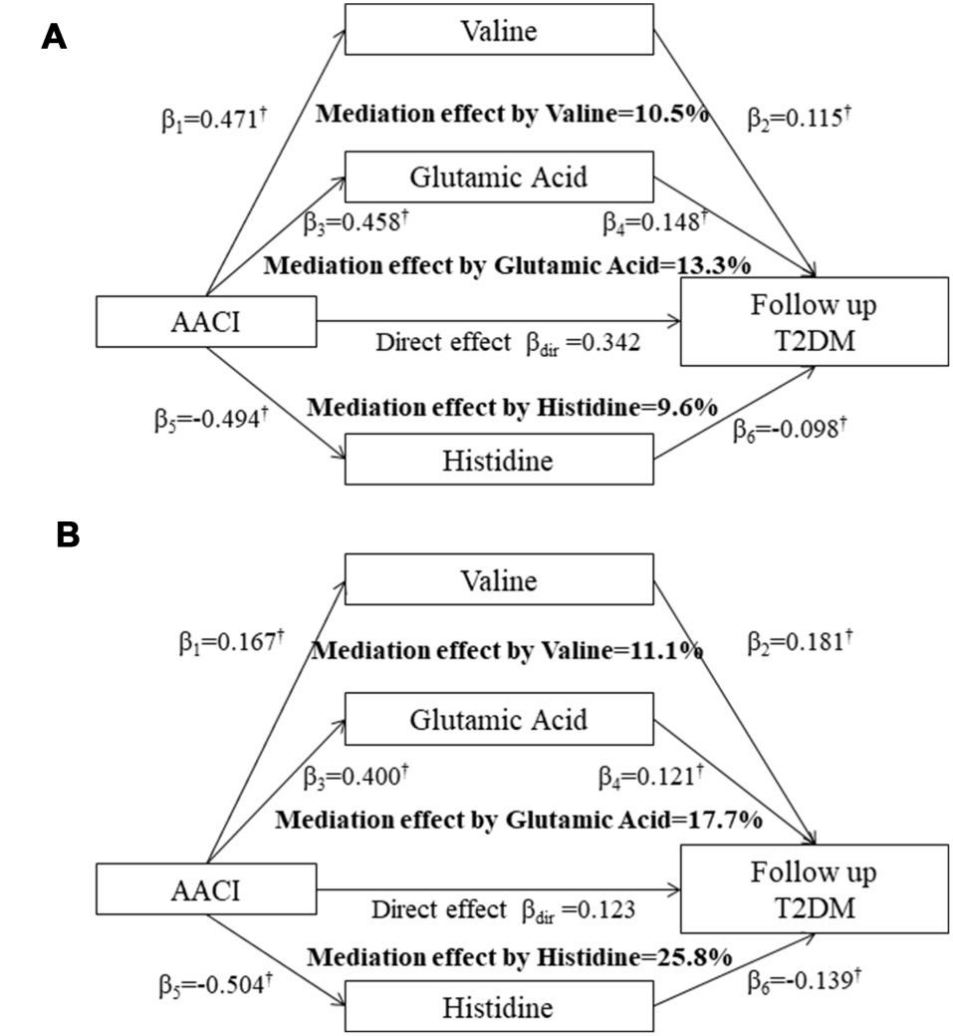
Mediation effects of serum valine, glutamic acid and histidine on the association between the AACI and incidence of type 2 diabetes in the HPHS (**A**) and HDNNCDS (**B**). AACI, dietary amino acids composition index; β, the standardised regression coefficients; ^†^ *P*<0.05 for the coefficients being different from 0. HPHS, Harbin People health Study; HDNNCDS, the Harbin Cohort Study on Diet, Nutrition and Chronic Noncommunicable Disease.

Figure 2 shows the mediation effects of the above three serum AAs on the association between AACI and T2DM in the two cohorts. The total effect of AACI on the risk of T2DM measured as standardized regression coefficient (β_tot_=0.512; *P*<0.001 in the HPHS; β_tot_=0.271; *P*<0.001 in the HDNNCDS) was estimated without the three serum AAs in the model with adjustment for covariates. The β_1_ to β_6_ were used to calculate the overall indirect effect for valine, glutamic acid and histidine respectively. The percentages of the total effect mediated by valine, glutamic acid and histidine were estimated at 10.5%, 13.3% and 9.6% in the HPHS, and 11.1%, 17.7% and 25.8% in the HDNNCDS.

### Food recommendations based on AACI

We conceptually grouped similar foods based on FFQ data, yielding 13 food types. Supplementary Table 3 displayed information on daily food intake, which included rice, wheat, potato, bean, vegetable, fruit, livestock, poultry, fish, egg, milk, snack and beverage from the HPHS and HDNNCDS cohorts. For each cohort, we conducted the statistical analyses of food intake information with tertiles of AACI. In the HPHS, the food of rice, wheat, potato, vegetable, fruit, livestock, poultry, fish, egg, milk, snack and beverage were significantly correlated with AACI. And rice had a significant positive correlation (*P*<0.001) to AACI, high AACI group tended to have a higher liking for rice. In contrast, other food intakes gradually decreased as the AACI increased. Consistent results were also observed in the HDNNCDS cohort.

## DISCUSSION

To our knowledge, this study is the first to address the link between dietary AAs composition and its serum profiles with the incidence of T2DM. To evaluate the overall quality of dietary AAs compositions, AACI was developed in this study by assessing the extent to which overall dietary AAs compositions adhered to the HRAAP. In the two prospective cohorts, the AACI was consistently and positively associated with six serum AAs and the incidence of T2DM. Among the six serum AAs, serum valine, glutamic acid and histidine consistently and partially mediated the association between the AACI and T2DM in the two cohorts.

In this study, using two prospective cohorts, a positive association between the AACI and incidence of T2DM was consistently observed, suggesting that participants with low quality of overall dietary essential AAs composition may have a higher risk of T2DM. Based on the concept of the HRAAP, the biological value of individual AAs can be influenced by overall dietary AAs compositions, resulting in different absorbed-, utilized- and metabolic-rates of dietary AAs [8–11]. Inadequate dietary AAs compositions therefore may play important roles in the development of T2DM. This study also demonstrated that when additionally adjusted for other known dietary risk factors for T2DM including intake of total protein [16], lipid [17], cholesterol [18], carbohydrate [19], fiber [20], saturated fat [21], fatty acids [22], and overall diet quality [23], the relationship between the AACI and incidence of T2DM was still significant, further indicating that inadequate dietary AAs composition was likely an important dietary factor for residual risk of T2DM. These findings are supported by previous studies, a population-based study showed that the risk of pre-diabetes varied with different dietary AAs patterns, which play an important role in glucose metabolism [24]. Imbalanced AAs components within dietary pattern had effects on liver fat accumulation, in which it was inflammation and oxidative stress that implicated in the evolution of the fatty liver disease and insulin resistance [25, 26]. Deviation from the ideal AAs patterns, inappropriate dietary AAs intakes could decrease the efficiency of amino acid utilization, meanwhile, resulting in increased plasma free fatty acids (FFA) which means increased levels of remaining nitrogen within the body [27]. Experimental evidence suggested that high nitrogen levels in the blood and elevated uric acid levels may increase insulin resistance and suppress insulin secretion [28]. They are also supported by cell and animal studies that feeding with a mixture of AAs rather than a single AA alone could promote the development of insulin-resistance and β-cell dysfunction in rodents [29, 30]. Taken together, dietary AAs composition may be an important factor to be considered in the prevention and management of T2DM.

Previous studies have reported that individual dietary AAs intake cannot commonly be reflected in serum AAs levels [3, 12, 13], which makes the current study of this issue lack compelling evidence for understanding the relationship between dietary AAs and T2DM. To fill this gap, this study further examined the association between the AACI and serum profiles of AAs in the two cohorts. The AACI was associated with eight serum AAs in the HPHS, and it was associated with nine serum AAs in the HDNNCDS. Although the difference of sample size and heterogeneity between the two cohorts are possible reasons for these discrepancy results, the serum valine, isoleucine, glycine, glutamic acid, phenylalanine and histidine were consistently observed to be associated with the AACI in the two cohorts, demonstrating that dietary AAs composition would influence the absorbed-, utilized- and metabolic-rates of AAs, which can be reflected in the serum AAs profiles, supporting the concept of the HRAAP for the impact of dietary AAs composition on their serum profiles. Studies regarding this issue were relatively scarce, but recent studies have reported that it is an overall dietary pattern rather than dietary BCAA was associated with serum BCAA, which partially supports the observations in this study [31, 32].

To further clarified whether and how the association between the AACI and incidence of T2DM mediated by serum AAs profiles for understanding the impact of dietary AAs composition on their serum profiles with subsequent T2DM, mediation analyses were performed in the two cohorts. Although the AACI was consistently associated with six serum AAs in the two cohorts, only serum valine, glutamic acid and histidine consistently and partially mediated this association in the two cohorts with total mediation effects of 33.4% and 54.6%, respectively, indicating that the association between inappropriate dietary AAs composition and increased risk of T2DM may be largely mediated by increasing serum valine and glutamic acid, and by decreasing serum histidine levels. Serum valine, as one of the BCAAs, has been consistently identified to be an important metabolite associated with insulin resistance in previous epidemiologic studies [33–37], probably through inhibition of insulin receptor substrate-1, and a recent study has reported that increased serum BCAAs probably produce more catabolic intermediates including propionyl CoA and succinyl CoA, resulting in accumulation of incompletely oxidized fatty acids and glucose [38]. Moreover, BCAAs can produce glutamic acids, catalyzing by branched-chain aminotransferase, and the glutamic acids have been reported to be associated with insulin resistance in the Framingham offspring study [39]. The potential protective effect of histidine on glucose/insulin homeostasis has been documented in previous research, probably by suppressing inflammatory factors and hepatic glucose production through central insulin action [40]. Importantly, evidence from Mendelian randomization analysis supports the association between BCAA-raising polymorphisms and a higher risk of T2DM, which could interpret the causal role of BCAA metabolism in T2DM risk [41]. Based on the findings in this study and these above mechanisms, this study therefore speculated that an inappropriate dietary AAs composition may cause varied absorbed-, utilized- and metabolic-rate of AAs, showing disordered plasma AAs profiles, and the dysregulated valine, glutamic acid and histidine may induce insulin resistance and subsequent diabetes.

Some mechanistic evidence supported the conclusion that inadequate dietary AAs composition could cause disturbed amino acid that is associated with the incidence of T2DM. Previous studies suggested that serum AAs act as nutrition signals, which have important effects on glucose homeostasis, insulin secretion from β-cells [42, 43]. AAs sensing signaling pathways included mechanistic target of rapamycin complex I (mTORC1) sensing AAs abundance, general control non-derepressible 2 (GCN2) sensing AAs deficiency in peripheral metabolic organs (such as pancreas, liver, adipose and muscle) [44]. Cultured β-cells studies confirmed that increased AAs drive and localize mTORC1 to lysosomal membrane, and promote mTORC1 activation, and phosphorylation of downstream effectors insulin receptor substrate 1 (IRS-1), contributing to IR [45–48]. Studies showed mTORC1 also has a potential role in the regulation of α cell glucagon secretion and glucose homeostasis [49]. Moreover, AAs may be involved in complex mechanisms via both adipocytes and hepatic cells. In the liver, extra AAs could lead to sustained activation of mTORC1, which enhances the expression of hepatic FFA, and inhibits FFA to TG conversion and autophagy, resulting in FFA lipotoxicity [50]. Similar results for adipocytes, elevated AAs activates AMPKα2 and stimulates lipolysis, which results in FFA accumulation [51]. Long-term exposure to elevated circulating FFA inhibits insulin signaling in the muscle, contributes to hyperglycemia in the liver, and decreases insulin secretion in the pancreas [52, 53]. Therefore, the above findings represent the potential key role of AA metabolism in the pathogenesis of diabetes and inappropriate AAs profiles could contribute to diabetes.

We conducted dietary behavioral analyses to find the right food through a limited number of food categories, and found high AACI group which is related to a high risk of developing diabetes tends to eat more rice. We could infer that rice amino acid components might be positively associated with the risk of T2DM. From the viewpoint of amino acids, rice as a major source of carbohydrates is low in protein that affected branched-chain and aromatic amino acid intakes [54]. Additionally, rice contains very low level of both lysine and threonine, which is associated with an increased risk of T2DM [55–57]. There is currently no amino acid evidence in terms of rice to support recommendations for the optimal prevention of T2DM. And, it could be hard for the rice to be separated from the overall diet with specific and clear recommendations for consumption within food guidelines. Further research is needed, with a deeper understanding relationship between food sources amino acids and T2DM.

Previous studies regarding this issue have frequently focused on the association between individual AA and T2DM, few studies have considered the overall quality of dietary AAs compositions. This study demonstrated that inadequate dietary AAs composition was associated with an increased incidence of T2DM, and the association between the AACI and serum AAs profiles and the potential mediation effects further strength our findings, which would improve our understanding of the pathobiology and mechanisms of T2DM, and facilitate selection of potential therapeutic and intervention strategies for T2DM. Moreover, the findings of this study also emphasized that future studies regarding dietary AAs and T2DM should consider dietary AAs as a whole rather than isolating individual AAs from diet in the prevention and management of T2DM.

The strength of our study is that it included two prospective cohorts with a relatively large OGTT sample of nutritional and metabolic analyses in this issue. Further, this study established the AACI based on the concept of the HRAAP for evaluating the overall quality of dietary AAs composition, demonstrated and emphasized the importance of dietary AAs composition. Third, the observed association between the AACI and T2DM was robust because it was observed in the two independent cohorts and it persisted after adjustment for a wide range of available confounding factors. However, we also recognize that our study has certain limitations. First, the study was observational in nature, and we cannot rule out the influence of unmeasured confounding factors. Besides, no amount of adjustment can deal completely with confounding in an observational context. Second, this study only included Asian subjects, which is likely to limit the generalizability of our findings to other ethnic populations. However, given the roles of HRAAP, and the association between serum AAs and T2DM has been shown be generally consistent across different ethnics. We would therefore expect that our observations would hold across other populations. Third, we also recognized that no mechanistic investigation is also the main drawback of this study. This study mainly focused on the population based on the relationship between dietary AA pattern and incidence of T2DM, emphasizing the importance of dietary AA pattern rather than individual AA in the development of T2DM; however, the future study based on experiments is warranted to validate these observations and provide more evidence in this issue. Finally, we acknowledge the limitations of the FFQ. Our AAs consumptions assessment relied on FFQ, was self-reported, data from FFQ may be subjected to recall bias and social desirability biases. Therefore, using FFQ as an instrument for quantifying AA consumptions is not sufficiently accurate. However, for the studies of assessing long-term dietary intake in large-scale epidemiological cohorts, food frequency questionnaires have proved useful and practical and this is the best way we can currently take [58].

In conclusion, this study demonstrated that dietary AAs composition was associated with the incidence of T2DM, which was likely responsible for the residual risk of classic known dietary factors for T2DM. Further, dietary AAs composition was associated with serum profiles of AAs, and serum valine, glutamic acid and histidine partially mediated the association between the inadequate dietary AAs composition and increased risk of T2DM. These findings may have important implications for the possible therapeutic and intervention strategies of T2DM.

## MATERIALS AND METHODS

### Study population

Two prospective study cohorts were recruited in Harbin, China, to investigate the impact of diet and nutrition on chronic non-communicable disease. They were the Harbin People health Study (HPHS) and the Harbin Cohort Study on Diet, Nutrition and Chronic Noncommunicable Disease (HDNNCDS) (registered at http://www.chictr.org as ChiCTR-ECH-12002721). Participants in the HPHS and HDNNCDS were recruited in 2008 and 2010, and the first in-person follow-up survey was completed in 2012 and 2016, with mean of 4.2 and 5.3 years follow-up. Detailed information of the two cohorts was described elsewhere [59, 60]. Briefly, a total of 1750 participants in the HPHS and 4024 participants in the HDNNCDS aged 20-74 years old who finished the baseline survey, measured fasting serum profiles of amino acids, were free of diabetes, and had calorie intake ranging from 500-4500 kcal/day at baseline were included in this study.

The two cohort studies were approved by the ethics committee of Harbin Medical University. The investigations were conducted in accordance with the Declaration of Helsinki, and written informed consent was provided by all participants. The methods in this study were performed in accordance with approved guidelines.

### Questionnaire survey

Detailed in-person interviews were administered by trained personnel using a structured questionnaire to collect information on demographic characteristics, lifestyles, physical condition and anthropometric characteristics in the two cohorts. Current smokers were defined as those who smoked at least 100 cigarettes in a lifetime or smoked every day or currently smoked some days. Current drinkers were defined as those who consumed ≥ 1 alcoholic drink each month in the 12 months before the survey. Regular exercise was defined as any kind of recreational or sport physical activity other than walking for work or life performed at least 30 minutes for three or more days per week. A family history of diabetes was defined as diabetes in first- or second-degree relatives.

### Dietary information

Dietary habits were recorded through food frequency questionnaires (FFQ). Before dietary surveys, two random subgroups of residents were recruited and were asked to complete two FFQs (FFQ1 and FFQ2) and a 3-day dietary record (DR) to validate the reliability of the FFQ. There was satisfactory consistency between two FFQs and the DR, indicating the FFQ is a reliable method for assessing dietary intakes [60]. The FFQ covered 103 food items assigned into 14 food groups: rice, wheaten foods, potato and its products, beans and its products, vegetables, fruits, livestock and its products, poultry and its products, dairy and its products, eggs and its products, fish and its products, snacks, beverage, and ice cream. The frequency and amount of each food item were recorded to calculate foods and nutrients intakes. According to the nutrient contents in the Chinese Food Composition Table [61], the nine essential dietary amino acids and two conditionally essential amino acids including isoleucine, leucine, lysine, methionine, phenylalanine, threonine, tryptophan, valine, histidine, cysteine and tyrosine were calculated by summing the amounts from each food item. The Alternate Healthy Eating Index (AHEI) was calculated and used to assess the overall diet quality [62].

### Development of AACI

The AACI was developed mainly based on the HRAAP reported by World Health Organization in 2007 [15]. The AACI was developed in two steps. First, the ratios between eleven amino acids including isoleucine, leucine, lysine, methionine, phenylalanine, threonine, valine, histidine, tryptophan, cysteine and tyrosine were calculated for deriving the composition of each AAs in the HRAAP (β_ratio_s).

$$\begin{array}{l} {\text{β}}_{\text{ratio}}\text{s}=\text{dietary}\;\text{AA}\;\text{levels}\;\text{based}\;\text{on}\;\text{HRAAP}\\ \;\;\;\;\;\;\;\;\;\;\;÷\text{dietary}\;\text{tryptophan}\;\text{levels}\;\text{based}\;\text{on}\;\text{HRAAP} \end{array}$$ (1)

Similarly, the ratios between eleven dietary amino acid intakes and dietary tryptophan intake were calculated for deriving the actual composition of these AAs in the diet. The satisfaction levels of the composition of each AAs adherence to the HRAAP were calculated based on the following equation:

$$\begin{array}{l} {\text{q}}_{\text{i}}=|(\text{actual}\;\text{dietary}\;\text{AA}\;\text{levels}\;\text{/}\\ \;\;\;\;\;\;\text{actual}\;\text{dietary}\;\text{tryptophan}\;\text{levels})\times (1/{\text{β}}_{\text{ratio}}\text{s})-1| \end{array}$$ (2)

Second, the sum of satisfaction levels of each AA was calculated for the AACI, indicating the extent that overall dietary amino acid composition adherence to the HRAAP. The lower the AACI, the more adherence the subject followed the HRAAP.

$$\text{AACI}=\sum_{i=1}^{11}{\text{q}}_{\text{i}}$$ (3)

### Anthropometric measurements and biochemical analyses

Anthropometric measurements, including height, weight, and waist circumference, were obtained by well-trained examiners, with the participants wearing light, thin clothing, and no shoes. Body weight and height were measured to the nearest 0.1 kg and 0.1 cm, respectively. Body mass index (BMI) was calculated as weight (kg) divided by the square of the height in meters (m^2^). An oral glucose tolerance test was performed in the two cohorts, according to the World Health Organization guidelines, for each subject. Serum glucose, triglyceride (TG), total cholesterol (TC), high-density lipoprotein cholesterol (HDL-C) and low-density lipoprotein cholesterol (LDL-C) were determined by an automatic analyzer (Hitachi 7100, Tokyo, Japan). Serum insulin was measured by Chemiluminescence Immune Analyzer. Glycosylated hemoglobin (HbA1c) was determined by high performance liquid chromatography (BIO-RAD VARIANT 2, USA). Homeostasis assessment model for IR was used to estimate hepatic IR (HOMA2-IR) with HOMA2 calculator updated by the University of Oxford in 2004, which is available from https://www.dtu.ox.ac.uk/homacalculator/.

### Serum amino acids measurement

Serum preparation for AAs quantitation was carried out as previously described [63]. Targeted analysis of serum amino acid profiles was performed by a Waters ACQUITY Ultra performance liquid chromatography (UPLC) system (Waters Corporation, Milford, MA) coupled to a Waters Xevo TQD mass spectrometer (MS) (Waters Corporation, Manchester, U.K.). The methods of UPLC and MS were described and validated in a previous study. Eighteen AAs, including threonine, glutamine, arginine, valine, leucine, isoleucine, phenylalanine, tryptophan, serine, methionine, glycine, proline, histidine, alanine, lysine, glutamic acid, aspartic acid and tyrosine, were determined in this study.

### Outcome measures

Type 2 diabetes was identified by self-reports of a history of diabetes diagnosis, and/or fasting blood glucose ≥ 7.0mmol/L, and/or 2-h glucose ≥ 11.1mmol/L, and/or receiving diabetes treatment. Incident type 2 diabetes cases were 385 in the HDNNCDS and 185 in the HPHS.

### Statistical analysis

All statistical analyses were performed in the R version 3.0.3 (http://www.r-project.org/), all *P*-values were two-tailed and *P*<0.05 was considered statistically significant. Baseline characteristics are presented as mean (SD) for continuous variables and percentages for categorical variables. For AACI and single intake of dietary AAs, the cutoff points were calculated. The AACI were categorized by tertiles, and the lowest tertile was used as the reference category. Baseline characteristics were compared using one-way ANOVA for continuous variables and the chi-square test for categorical variables across tertiles of the AACI. Logistic regression models were performed to examine the association between the tertiles of AACI and incidence of T2DM. Linear regression was used to explore the association between the AACI and profiles of serum AAs levels. Once the association between the AACI and serum AAs levels had been confirmed, mediation models were constructed to examine whether and how the association of the AACI with future risk of T2DM was mediated by serum AAs using R package *Lavaan* [64].

## Supplementary Material

Supplementary Tables

## References

[1] Bloomgarden Z. Diabetes and branched-chain amino acids: what is the link? J Diabetes. 2018; 10:350–52. 10.1111/1753-0407.12645 29369529

[2] Gar C, Rottenkolber M, Prehn C, Adamski J, Seissler J, Lechner A. Serum and plasma amino acids as markers of prediabetes, insulin resistance, and incident diabetes. Crit Rev Clin Lab Sci. 2018; 55:21–32. 10.1080/10408363.2017.1414143 29239245

[3] Jennings A, MacGregor A, Pallister T, Spector T, Cassidy A. Associations between branched chain amino acid intake and biomarkers of adiposity and cardiometabolic health independent of genetic factors: A twin study. Int J Cardiol. 2016; 223:992–98. 10.1016/j.ijcard.2016.08.307 27591698 PMC5074005

[4] Nagata C, Nakamura K, Wada K, Tsuji M, Tamai Y, Kawachi T. Branched-chain amino acid intake and the risk of diabetes in a Japanese community: the Takayama study. Am J Epidemiol. 2013; 178:1226–32. 10.1093/aje/kwt112 24008908

[5] van Loon LJ, Kruijshoop M, Menheere PP, Wagenmakers AJ, Saris WH, Keizer HA. Amino acid ingestion strongly enhances insulin secretion in patients with long-term type 2 diabetes. Diabetes Care. 2003; 26:625–30. 10.2337/diacare.26.3.625 12610012

[6] Argyrakopoulou G, Kontrafouri P, Eleftheriadou I, Kokkinos A, Arapostathi C, Kyriaki D, Perrea D, Revenas C, Katsilambros N, Tentolouris N. The effect of the oral administration of leucine on endothelial function, glucose and insulin concentrations in healthy subjects. Exp Clin Endocrinol Diabetes. 2019; 127:505–10. 10.1055/a-0597-8985 29890542

[7] Leenders M, Verdijk LB, van der Hoeven L, van Kranenburg J, Hartgens F, Wodzig WK, Saris WH, van Loon LJ. Prolonged leucine supplementation does not augment muscle mass or affect glycemic control in elderly type 2 diabetic men. J Nutr. 2011; 141:1070–76. 10.3945/jn.111.138495 21525248

[8] Moore DR, Soeters PB. The biological value of protein. Nestle Nutr Inst Workshop Ser. 2015; 82:39–51. 10.1159/000382000 26545252

[9] Albanese AA, Irby V. Observations on the biological value of a mixture of essential amino acids. Science. 1943; 98:286–88. 10.1126/science.98.2543.286 17811501

[10] Acevedo-Pacheco L, Serna-Saldívar SO. In vivo protein quality of selected cereal-based staple foods enriched with soybean proteins. Food Nutr Res. 2016; 60:31382. 10.3402/fnr.v60.31382 27765143 PMC5073300

[11] Jonker R, Engelen MP, Deutz NE. Role of specific dietary amino acids in clinical conditions. Br J Nutr. 2012 (Suppl 2); 108:S139–48. 10.1017/S0007114512002358 23107525 PMC4734127

[12] McCormack SE, Shaham O, McCarthy MA, Deik AA, Wang TJ, Gerszten RE, Clish CB, Mootha VK, Grinspoon SK, Fleischman A. Circulating branched-chain amino acid concentrations are associated with obesity and future insulin resistance in children and adolescents. Pediatr Obes. 2013; 8:52–61. 10.1111/j.2047-6310.2012.00087.x 22961720 PMC3519972

[13] Schmidt JA, Rinaldi S, Scalbert A, Ferrari P, Achaintre D, Gunter MJ, Appleby PN, Key TJ, Travis RC. Plasma concentrations and intakes of amino acids in male meat-eaters, fish-eaters, vegetarians and vegans: a cross-sectional analysis in the EPIC-Oxford cohort. Eur J Clin Nutr. 2016; 70:306–12. 10.1038/ejcn.2015.144 26395436 PMC4705437

[14] Zheng Y, Li Y, Qi Q, Hruby A, Manson JE, Willett WC, Wolpin BM, Hu FB, Qi L. Cumulative consumption of branched-chain amino acids and incidence of type 2 diabetes. Int J Epidemiol. 2016; 45:1482–92. 10.1093/ije/dyw143 27413102 PMC5100612

[15] Joint WHO/FAO/UNU Expert Consultation. Protein and amino acid requirements in human nutrition. World Health Organ Tech Rep Ser. 2007;235:1–265. 18330140

[16] van Nielen M, Feskens EJ, Mensink M, Sluijs I, Molina E, Amiano P, Ardanaz E, Balkau B, Beulens JW, Boeing H, Clavel-Chapelon F, Fagherazzi G, Franks PW, et al, and InterAct Consortium. Dietary protein intake and incidence of type 2 diabetes in Europe: the EPIC-InterAct Case-Cohort Study. Diabetes Care. 2014; 37:1854–62. 10.2337/dc13-2627 24722499

[17] Rice Bradley BH. Dietary fat and risk for type 2 diabetes: a review of recent research. Curr Nutr Rep. 2018; 7:214–26. 10.1007/s13668-018-0244-z 30242725 PMC6244743

[18] Baghdasarian S, Lin HP, Pickering RT, Mott MM, Singer MR, Bradlee ML, Moore LL. Dietary cholesterol intake is not associated with risk of type 2 diabetes in the framingham offspring study. Nutrients. 2018; 10:665. 10.3390/nu10060665 29794966 PMC6024792

[19] AlEssa HB, Bhupathiraju SN, Malik VS, Wedick NM, Campos H, Rosner B, Willett WC, Hu FB. Carbohydrate quality and quantity and risk of type 2 diabetes in US women. Am J Clin Nutr. 2015; 102:1543–53. 10.3945/ajcn.115.116558 26537938 PMC4658465

[20] InterAct Consortium. Dietary fibre and incidence of type 2 diabetes in eight European countries: the EPIC-InterAct Study and a meta-analysis of prospective studies. Diabetologia. 2015; 58:1394–408. 10.1007/s00125-015-3585-9 26021487 PMC4472947

[21] Risérus U, Willett WC, Hu FB. Dietary fats and prevention of type 2 diabetes. Prog Lipid Res. 2009; 48:44–51. 10.1016/j.plipres.2008.10.002 19032965 PMC2654180

[22] Brown TJ, Brainard J, Song F, Wang X, Abdelhamid A, Hooper L, and PUFAH Group. Omega-3, omega-6, and total dietary polyunsaturated fat for prevention and treatment of type 2 diabetes mellitus: systematic review and meta-analysis of randomised controlled trials. BMJ. 2019; 366:l4697. 10.1136/bmj.l4697 31434641 PMC6699594

[23] Ley SH, Pan A, Li Y, Manson JE, Willett WC, Sun Q, Hu FB. Changes in overall diet quality and subsequent type 2 diabetes risk: three U.S. Diabetes Care. 2016; 39:2011–18. 10.2337/dc16-0574 27634391 PMC5079614

[24] Mirmiran P, Bahadoran Z, Esfandyari S, Azizi F. Dietary protein and amino acid profiles in relation to risk of dysglycemia: findings from a prospective population-based study. Nutrients. 2017; 9:971. 10.3390/nu9090971 28869547 PMC5622731

[25] Galarregui C, Cantero I, Marin-Alejandre BA, Monreal JI, Elorz M, Benito-Boillos A, Herrero JI, de la O V, Ruiz-Canela M, Hermsdorff HH, Bressan J, Tur JA, Martínez JA, et al. Dietary intake of specific amino acids and liver status in subjects with nonalcoholic fatty liver disease: fatty liver in obesity (FLiO) study. Eur J Nutr. 2020. [Epub ahead of print]. 10.1007/s00394-020-02370-6 32857176

[26] Zhang F, Zhao S, Yan W, Xia Y, Chen X, Wang W, Zhang J, Gao C, Peng C, Yan F, Zhao H, Lian K, Lee Y, et al. Branched chain amino acids cause liver injury in obese/diabetic mice by promoting adipocyte lipolysis and inhibiting hepatic autophagy. EBioMedicine. 2016; 13:157–67. 10.1016/j.ebiom.2016.10.013 27843095 PMC5264279

[27] Alvarez FJ, Ryman K, Hooijmaijers C, Bulone V, Ljungdahl PO. Diverse nitrogen sources in seminal fluid act in synergy to induce filamentous growth of Candida albicans. Appl Environ Microbiol. 2015; 81:2770–80. 10.1128/AEM.03595-14 25662979 PMC4375319

[28] Xie Y, Bowe B, Li T, Xian H, Yan Y, Al-Aly Z. Higher blood urea nitrogen is associated with increased risk of incident diabetes mellitus. Kidney Int. 2018; 93:741–52. 10.1016/j.kint.2017.08.033 29241622

[29] Newgard CB, An J, Bain JR, Muehlbauer MJ, Stevens RD, Lien LF, Haqq AM, Shah SH, Arlotto M, Slentz CA, Rochon J, Gallup D, Ilkayeva O, et al. A branched-chain amino acid-related metabolic signature that differentiates obese and lean humans and contributes to insulin resistance. Cell Metab. 2009; 9:311–26. 10.1016/j.cmet.2009.02.002 19356713 PMC3640280

[30] Zhang Y, Guo K, LeBlanc RE, Loh D, Schwartz GJ, Yu YH. Increasing dietary leucine intake reduces diet-induced obesity and improves glucose and cholesterol metabolism in mice via multimechanisms. Diabetes. 2007; 56:1647–54. 10.2337/db07-0123 17360978

[31] Merz B, Frommherz L, Rist MJ, Kulling SE, Bub A, Watzl B. Dietary pattern and plasma BCAA-variations in healthy men and women-results from the KarMeN study. Nutrients. 2018; 10:623. 10.3390/nu10050623 29762522 PMC5985475

[32] Rousseau M, Guénard F, Garneau V, Allam-Ndoul B, Lemieux S, Pérusse L, Vohl MC. Associations between dietary protein sources, plasma BCAA and short-chain acylcarnitine levels in adults. Nutrients. 2019; 11:173. 10.3390/nu11010173 30650556 PMC6356602

[33] Lee CC, Watkins SM, Lorenzo C, Wagenknecht LE, Il’yasova D, Chen YD, Haffner SM, Hanley AJ. Branched-chain amino acids and insulin metabolism: the insulin resistance atherosclerosis study (IRAS). Diabetes Care. 2016; 39:582–88. 10.2337/dc15-2284 26895884 PMC4806771

[34] Würtz P, Tiainen M, Mäkinen VP, Kangas AJ, Soininen P, Saltevo J, Keinänen-Kiukaanniemi S, Mäntyselkä P, Lehtimäki T, Laakso M, Jula A, Kähönen M, Vanhala M, Ala-Korpela M. Circulating metabolite predictors of glycemia in middle-aged men and women. Diabetes Care. 2012; 35:1749–56. 10.2337/dc11-1838 22563043 PMC3402262

[35] Wang TJ, Larson MG, Vasan RS, Cheng S, Rhee EP, McCabe E, Lewis GD, Fox CS, Jacques PF, Fernandez C, O’Donnell CJ, Carr SA, Mootha VK, et al. Metabolite profiles and the risk of developing diabetes. Nat Med. 2011; 17:448–53. 10.1038/nm.2307 21423183 PMC3126616

[36] Tai ES, Tan ML, Stevens RD, Low YL, Muehlbauer MJ, Goh DL, Ilkayeva OR, Wenner BR, Bain JR, Lee JJ, Lim SC, Khoo CM, Shah SH, Newgard CB. Insulin resistance is associated with a metabolic profile of altered protein metabolism in Chinese and Asian-Indian men. Diabetologia. 2010; 53:757–67. 10.1007/s00125-009-1637-8 20076942 PMC3753085

[37] Guasch-Ferré M, Hruby A, Toledo E, Clish CB, Martínez-González MA, Salas-Salvadó J, Hu FB. Metabolomics in prediabetes and diabetes: a systematic review and meta-analysis. Diabetes Care. 2016; 39:833–46. 10.2337/dc15-2251 27208380 PMC4839172

[38] Newgard CB. Interplay between lipids and branched-chain amino acids in development of insulin resistance. Cell Metab. 2012; 15:606–14. 10.1016/j.cmet.2012.01.024 22560213 PMC3695706

[39] Cheng S, Rhee EP, Larson MG, Lewis GD, McCabe EL, Shen D, Palma MJ, Roberts LD, Dejam A, Souza AL, Deik AA, Magnusson M, Fox CS, et al. Metabolite profiling identifies pathways associated with metabolic risk in humans. Circulation. 2012; 125:2222–31. 10.1161/CIRCULATIONAHA.111.067827 22496159 PMC3376658

[40] Kimura K, Nakamura Y, Inaba Y, Matsumoto M, Kido Y, Asahara S, Matsuda T, Watanabe H, Maeda A, Inagaki F, Mukai C, Takeda K, Akira S, et al. Histidine augments the suppression of hepatic glucose production by central insulin action. Diabetes. 2013; 62:2266–77. 10.2337/db12-1701 23474485 PMC3712067

[41] Lotta LA, Scott RA, Sharp SJ, Burgess S, Luan J, Tillin T, Schmidt AF, Imamura F, Stewart ID, Perry JR, Marney L, Koulman A, Karoly ED, et al. Genetic predisposition to an impaired metabolism of the branched-chain amino acids and risk of type 2 diabetes: a mendelian randomisation analysis. PLoS Med. 2016; 13:e1002179. 10.1371/journal.pmed.1002179 27898682 PMC5127513

[42] Lynch CJ, Adams SH. Branched-chain amino acids in metabolic signalling and insulin resistance. Nat Rev Endocrinol. 2014; 10:723–36. 10.1038/nrendo.2014.171 25287287 PMC4424797

[43] Jewell JL, Russell RC, Guan KL. Amino acid signalling upstream of mTOR. Nat Rev Mol Cell Biol. 2013; 14:133–39. 10.1038/nrm3522 23361334 PMC3988467

[44] Hu X, Guo F. Amino acid sensing in metabolic homeostasis and health. Endocr Rev. 2020. [Epub ahead of print]. 10.1210/endrev/bnaa026 33053153

[45] Sancak Y, Peterson TR, Shaul YD, Lindquist RA, Thoreen CC, Bar-Peled L, Sabatini DM. The Rag GTPases bind raptor and mediate amino acid signaling to mTORC1. Science. 2008; 320:1496–501. 10.1126/science.1157535 18497260 PMC2475333

[46] Kim E, Goraksha-Hicks P, Li L, Neufeld TP, Guan KL. Regulation of TORC1 by Rag GTPases in nutrient response. Nat Cell Biol. 2008; 10:935–45. 10.1038/ncb1753 18604198 PMC2711503

[47] Tremblay F, Brûlé S, Hee Um S, Li Y, Masuda K, Roden M, Sun XJ, Krebs M, Polakiewicz RD, Thomas G, Marette A. Identification of IRS-1 Ser-1101 as a target of S6K1 in nutrient- and obesity-induced insulin resistance. Proc Natl Acad Sci USA. 2007; 104:14056–61. 10.1073/pnas.0706517104 17709744 PMC1950339

[48] Nie C, He T, Zhang W, Zhang G, Ma X. Branched Chain Amino Acids: Beyond Nutrition Metabolism. Int J Mol Sci. 2018; 19:954. 10.3390/ijms19040954 29570613 PMC5979320

[49] Hayashi Y, Seino Y. Regulation of amino acid metabolism and α-cell proliferation by glucagon. J Diabetes Investig. 2018; 9:464–72. 10.1111/jdi.12797 29314731 PMC5934249

[50] Efeyan A, Zoncu R, Chang S, Gumper I, Snitkin H, Wolfson RL, Kirak O, Sabatini DD, Sabatini DM. Regulation of mTORC1 by the Rag GTPases is necessary for neonatal autophagy and survival. Nature. 2013; 493:679–83. 10.1038/nature11745 23263183 PMC4000705

[51] Wu Y, Song P, Zhang W, Liu J, Dai X, Liu Z, Lu Q, Ouyang C, Xie Z, Zhao Z, Zhuo X, Viollet B, Foretz M, et al. Activation of AMPKα2 in adipocytes is essential for nicotine-induced insulin resistance *in vivo*. Nat Med. 2015; 21:373–82. 10.1038/nm.3826 25799226 PMC4390501

[52] Chen X, Scholl TO, Leskiw M, Savaille J, Stein TP. Differences in maternal circulating fatty acid composition and dietary fat intake in women with gestational diabetes mellitus or mild gestational hyperglycemia. Diabetes Care. 2010; 33:2049–54. 10.2337/dc10-0693 20805277 PMC2928361

[53] Boden G, Shulman GI. Free fatty acids in obesity and type 2 diabetes: defining their role in the development of insulin resistance and beta-cell dysfunction. Eur J Clin Invest. 2002 (Suppl 3); 32:14–23. 10.1046/j.1365-2362.32.s3.3.x 12028371

[54] Kohlmeier M. Nutrient Metabolism. Elsevier’s science. 2003.

[55] Duran-Jimenez B, Dobler D, Moffatt S, Rabbani N, Streuli CH, Thornalley PJ, Tomlinson DR, Gardiner NJ. Advanced glycation end products in extracellular matrix proteins contribute to the failure of sensory nerve regeneration in diabetes. Diabetes. 2009; 58:2893–903. 10.2337/db09-0320 19720799 PMC2780874

[56] Galili G, Karchi H, Shaul O, Perl A, Cahana A, Tzchori IB, Zhu XZ, Galili S. Production of transgenic plants containing elevated levels of lysine and threonine. Biochem Soc Trans. 1994; 22:921–25. 10.1042/bst0220921 7698485

[57] Tessari P, Cecchet D, Cosma A, Vettore M, Coracina A, Millioni R, Iori E, Puricelli L, Avogaro A, Vedovato M. Nitric oxide synthesis is reduced in subjects with type 2 diabetes and nephropathy. Diabetes. 2010; 59:2152–59. 10.2337/db09-1772 20484137 PMC2927936

[58] Willett W. Nutritional Epidemiology. 3rd edition. Oxford University Press. 2012. 10.1093/acprof:oso/9780199754038.001.0001

[59] Na L, Wu X, Feng R, Li J, Han T, Lin L, Lan L, Yang C, Li Y, Sun C. The harbin cohort study on diet, nutrition and chronic non-communicable diseases: study design and baseline characteristics. PLoS One. 2015; 10:e0122598. 10.1371/journal.pone.0122598 25856294 PMC4391912

[60] Du S, Wu X, Han T, Duan W, Liu L, Qi J, Niu Y, Na L, Sun C. Dietary manganese and type 2 diabetes mellitus: two prospective cohort studies in China. Diabetologia. 2018; 61:1985–95. 10.1007/s00125-018-4674-3 29971528

[61] Yang YW, Pan X. China Food Composition Tables. China, Beijing University Medical Press. 2009.

[62] Wang DD, Leung CW, Li Y, Ding EL, Chiuve SE, Hu FB, Willett WC. Trends in dietary quality among adults in the United States, 1999 through 2010. JAMA Intern Med. 2014; 174:1587–95. 10.1001/jamainternmed.2014.3422 25179639 PMC5924699

[63] Liu L, Feng R, Guo F, Li Y, Jiao J, Sun C. Targeted metabolomic analysis reveals the association between the postprandial change in palmitic acid, branched-chain amino acids and insulin resistance in young obese subjects. Diabetes Res Clin Pract. 2015; 108:84–93. 10.1016/j.diabres.2015.01.014 25700627

[64] Rosseel Y, lavaan RY. An R Package for Structural Equation Modeling. J Stat Softw. 2012; 48:1-36. 10.18637/jss.v048.i02

