# Supplementary material for: Extent reflecting overall dietary amino acids composition adherence to the human requirement amino acids pattern is associated with the development of type 2 diabetes
Source: Aging (Albany NY). 2021 Mar 26;13(7):10141–57. doi: 10.18632/aging.202777 (PMC8064212; doi:10.18632/aging.202777)
Supplement: Supplementary Tables [file aging-13-202777-s001.pdf]

SUPPLEMENTARY TABLES

Supplementary Table 1. Baseline characteristics of participants in the HPHS and HDNNCDS by type 2 diabetes.

	HDNNCDS				HPHS				
Variable	Without diabetes (N=3639)	Diabetes (N=385)	*P*-value	Total (N=4024)	Without diabetes (N=1601)	Diabetes (N=148)	*P*-value	Total (N=1749)	*P*-value*
Age (years)	49.3(9.7)	52.0(9.1)	<0.001	49.6(9.6)	45.6(10.4)	49.4(10.7)	<0.001	45.9(10.4)	<0.001
Male [n, (%)]	1183(32.5)	157(40.8)	0.001	1340(33.3)	487(30.4)	56(37.8)	<0.001	544(31.1)	0.099
Alcohol consumption [n, (%)]	1277(35.1)	136(35.3)	0.955	1413(35.1)	459(28.7)	43(29.1)	0.924	503(28.8)	<0.001
Current smoking [n, (%)]	559(15.4)	63(16.4)	0.817	622(15.5)	232(14.5)	26(17.6)	0.135	258(14.8)	0.773
Regular exercise habits [n, (%)]	1968(54.1)	176(45.7)	<0.001	2144(53.3)	900(56.2)	91(61.5)	0.226	991(56.7)	0.018
Family history of diabetes [n (%)]	529(14.5)	71(18.4)	0.050	600(14.9)	163(10.2)	28(18.9)	0.618	191(10.9)	0.001
Over senior middle school [n (%)]	2639(72.5)	260(67.5)	0.042	2899(72.0)	1023(63.9)	80(54.1)	0.013	1103 (63.1)	<0.001
BMI (kg/m^2)	24.7(3.4)	26.0(3.4)	<0.001	24.9(3.5)	25.0(3.4)	26.6(4.1)	<0.001	25.2(3.5)	<0.001
Total calorie intake (kcal/d)	2377(887)	2399(914)	0.914	2380(890)	2211(880)	2258(843)	0.518	2253(847)	<0.001
Protein (g/day)	72.4(36.9)	73.3(35.4)	0.800	72.5(36.8)	68.3(33.7)	65.3(30.8)	0.264	68.0(33.4)	<0.001
Fiber (g/day)	14.1(6.9)	13.7(7.3)	0.231	14.0(7.0)	14.3(7.3)	13.6(6.8)	0.180	14.2(7.2)	0.112
Saturated fatty acid (g/day)	16.2(8.4)	16.6(7.9)	0.246	16.2(8.3)	14.1(6.7)	15.1(7.1)	0.105	14.9(6.7)	<0.001
Fasting glucose (mmol/L)	4.43(0.59)	5.36(0.95)	<0.001	4.52(0.69)	4.67(0.67)	5.09(0.87)	<0.001	4.71(0.70)	<0.001
2-hour glucose (mmol/L)	5.63(1.47)	6.57(2.21)	<0.001	5.80(1.64)	5.60(1.57)	6.76(2.25)	<0.001	5.68(1.67)	0.790
Fasting insulin (μU/mL)	8.1(6.4)	12.2(21.1)	<0.001	8.5(6.1)	8.2(7.8)	10.5(14.9)	0.022	8.5(9.1)	0.744
TG (mmol/L)	1.64(1.57)	2.03(1.93)	<0.001	1.69(1.61)	1.69(1.26)	2.08(1.73)	0.026	1.73(1.31)	0.146
TCHO (mmol/L)	5.10(1.00)	5.31(1.03)	0.003	5.12(1.01)	4.90(0.94)	5.10(0.81)	0.131	4.91(0.93)	<0.001
HDL-C (mmol/L)	1.27(0.32)	1.18(0.31)	<0.001	1.26(0.32)	1.29(0.33)	1.28(0.35)	0.750	1.28(0.33)	0.041
LDL-C (mmol/L)	2.99(0.85)	3.11(0.87)	0.088	3.00(0.85)	2.87(0.97)	2.94(0.90)	0.573	2.87(0.97)	0.006

Mean ± Standard Deviation was used for continuous variables.
One-way ANOVA was used for continuous variables; Chi-square test was used for categorical variables.
BMI, body mass index; TG, triglyceride; TCHO, total cholesterol; HDL-C, high-density lipoprotein cholesterol; LDL-C, low-density lipoprotein cholesterol; HPHS, Harbin People health Study; HDNNCDS, the Harbin Cohort Study on Diet, Nutrition and Chronic Noncommunicable Disease.

Supplementary Table 2. Associations between serum AAs influenced by AACI and incidence of type 2 diabetes in the HPHS and HDNNCDS.

Serum AAs	Case/N	Model 1	Model 2	Model 3
**HDNNCDS**				
Valine				
<87.1	77/1010	1	1	1
87.1-98.1	64/1006	0.81(0.57-1.15)	0.82(0.58-1.16)	0.81(0.57-1.15)
98.1-108.7	78/1005	1.08(0.77-1.52)	1.07(0.76-1.50)	1.07(0.76-1.50)
>108.7	166/1003	2.52(1.86-3.41)	2.47(1.82-3.35)	2.36(1.73-3.21)
P for trend		<0.001	<0.001	<0.001
Isoleucine				
<17.3	80/1015	1	1	1
17.3-21.7	94/999	1.24(0.91-1.71)	1.26(0.92-1.74)	1.29(0.94-1.78)
21.7-27.1	87/1017	1.11(0.80-1.54)	1.13(0.81-1.56)	1.12(0.81-1.56)
>27.1	124/993	1.60(1.17-2.19)	1.62(1.18-2.22)	1.43(1.03-1.97)
P for trend		0.016	0.014	0.069
Glycine				
<259.5	122/1006	1	1	1
259.5-286.8	106/1006	0.84(0.63-1.11)	0.84(0.63-1.12)	0.89(0.66-1.18)
286.8-309.2	78/1006	0.57(0.41-0.78)	0.57(0.41-0.78)	0.59(0.43-0.81)
>309.2	79/1006	0.54(0.39-0.74)	0.54(0.39-0.75)	0.56(0.40-0.78)
P for trend		<0.001	<0.001	0.001
Glutamic acid				
<45.6	72/1007	1	1	1
45.6-51.2	56/1024	0.84(0.58-1.22)	0.83(0.58-1.20)	0.81(0.56-1.17)
51.2-56.0	111/996	1.41(1.03-1.93)	1.40(1.02-1.92)	1.35(0.98-1.86)
>56.0	146/997	1.78(1.29-2.44)	1.77(1.28-2.43)	1.75(1.26-2.41)
P for trend		<0.001	<0.001	0.003
Phenylalanine				
<45.6	88/1006	1	1	1
45.6-51.2	72/1013	0.80(0.58-1.12)	0.81(0.58-1.12)	0.83(0.60-1.16)
51.2-56.0	94/1000	1.22(0.89-1.66)	1.21(0.88-1.65)	1.24(0.90-1.70)
>56.0	131/1005	1.64(1.22-2.20)	1.62(1.21-2.19)	1.54(1.14-2.08)
P for trend		<0.001	<0.001	0.001
Histidine				
<45.6	132/1006	1	1	1
45.6-51.2	70/1007	0.78(0.53-1.03)	0.81(0.61-1.09)	0.85(0.61-1.19)
51.2-56.0	108/1006	0.58(0.26-0.92)	0.62(0.35-1.01)	0.63(0.42-1.04)
>56.0	75/1005	0.37(0.19-0.53)	0.39(0.22-0.75)	0.43(0.28-0.65)
P for trend		<0.001	<0.001	<0.001
**HPHS**				
Valine				
<69.2	26/439	1	1	1
69.2-85.5	29/436	1.76(0.82-3.82)	1.70(0.78-3.68)	1.71(0.79-3.71)
85.5-109.3	43/437	2.46(1.18-5.15)	2.28(1.09-4.79)	2.28(1.08-4.79)
>109.3	43/438	2.76(1.34-5.69)	2.57(1.24-5.32)	2.55(1.23-5.28)
P for trend		0.004	0.008	0.009
Isoleucine				
<25.2	21/438	1	1	1
25.2-33.9	31/437	1.45(0.80-2.61)	1.44(0.80-2.61)	1.37(0.76-2.49)
33.9-39.4	40/437	1.98(1.12-3.50)	1.91(1.08-3.38)	1.82(1.02-3.24)
>39.4	49/438	2.63(1.50-4.60)	2.42(1.38-4.24)	2.22(1.26-3.93)
P for trend		0.004	0.014	0.035
Glycine				
<252.8	36/437	1	1	1
252.8-285.6	43/437	1.24(0.77-1.99)	1.26(0.78-2.03)	1.25(0.77-2.02)
285.6-317.9	32/437	0.84(0.50-1.40)	0.84(0.51-1.41)	0.84(0.50-1.40)
>317.9	30/439	0.75(0.44-1.26)	0.77(0.46-1.31)	0.76(0.45-1.29)
P for trend		0.201	0.213	0.212

Glutamic acid				
<41.7	18/439	1	1	1
41.7-51.0	31/436	1.55(0.85-2.86)	1.51(0.82-2.78)	1.46(0.79-2.69)
51.0-67.9	45/437	2.60(1.47-4.60)	2.46(1.38-4.37)	2.34(1.31-4.17)
>56.0	47/438	2.71(1.53-4.78)	2.50(1.41-4.46)	2.46(1.38-4.37)
P for trend		0.001	0.003	0.005
Phenylalanine				
<45.6	36/439	1	1	1
45.6-51.2	30/436	0.76(0.45-1.26)	0.76(0.46-1.28)	0.76(0.46-1.28)
51.2-56.0	39/438	1.01(0.62-1.64)	1.02(0.63-1.66)	1.02(0.63-1.66)
>56.0	43/437	1.25(0.78-2.01)	1.21(0.75-1.94)	1.12(0.69-1.82)
P for trend		0.271	0.369	0.513
Histidine				
<52.5	38/437	1	1	1
52.5-58.2	37/438	0.72(0.44-1.17)	0.76(0.46-1.25)	0.76(0.46-1.25)
58.2-64.3	38/437	0.55(0.32-0.92)	0.60(0.35-1.03)	0.58(0.34-0.99)
>64.3	28/438	0.32(0.18-0.59)	0.36(0.20-0.67)	0.36(0.20-0.67)
P for trend		0.003	0.012	0.012

Data are RRs (95%CI).
Model 1 was adjusted by demographic covariates including age, gender, BMI, education, alcohol consumption rate, smoking rate and regular exercise habits;
Model 2 was further adjusted by nutritional covariates including dietary energy intake, protein intake, fiber, saturated fatty acid, overall diet quality and AACI;
Model 3 was further adjusted by biochemical indices including total cholesterol, triglyceride, high-density lipoprotein cholesterol, low-density lipoprotein cholesterol and HOMA2-IR.

Supplementary Table 3. Food information across tertiles of AACI in the HPHS and HDNNCDS.

Variable	HPHS				HDNNCDS			
	Tertile 1 (N=584)	Tertile 2 (N=582)	Tertile 3 (N=584)	*P*-value	Tertile 1 (N=1341)	Tertile 2 (N=1341)	Tertile 3 (N=1342)	*P*-value
Rice (g/day)	123.04±97.9	190.43±111.38	307.46±159.48	<0.001	122.02±71.9	169.11±76.66	266.37±110.05	<0.001
Wheat (g/day)	145.85±115.94	140.95±97.74	121.48±113.04	<0.001	124.89±124.35	126.54±143.42	104.79±162.5	<0.001
Potato (g/day)	67.81±81.84	56.71±55.28	49.33±60.29	<0.001	67.76±58.03	58.09±42.35	52.84±37.52	<0.001
Bean (g/day)	50.72±56.51	48.81±49.31	49.5±75.3	0.866	75.45±50.48	79.14±47.8	79.02±58.76	0.122
Vegetable (g/day)	302.74±214.94	306.67±204.19	267.24±256.93	0.005	276.33±124.51	281.61±134.42	242.38±122.91	<0.001
Fruit (g/day)	191.05±203.15	159.6±148.21	130.56±144.94	<0.001	149.96±107.38	139.89±85.21	119.41±84.57	<0.001
Livestock (g/day)	89.82±89.38	66.27±64.58	40.03±41.87	<0.001	86.68±64.77	72.24±50.99	54.61±36.68	<0.001
Poultry (g/day)	34.9±54.83	20.88±29.01	12.62±16.97	<0.001	27.51±40.49	17.19±21.52	12.05±12.96	<0.001
Fish (g/day)	64.98±133.23	20.4±22.38	13.58±15.26	<0.001	46.72±64.5	25.62±18.53	19.18±13.08	<0.001
Egg (g/day)	51.98±50.86	41.66±35.79	30.74±25.56	<0.001	49.22±43.1	39.55±20.83	33.99±20.21	<0.001
Milk (ml/day)	2.8±2.65	1.77±1.79	0.9±1.34	<0.001	2.88±2.05	2.23±1.67	1.57±1.4	<0.001
Snack (g/day)	20.95±38.86	12.33±28.38	6.63±12.85	<0.001	26.88±32.71	17.52±17.98	12.9±12.88	<0.001
Beverage (g/day)	0.95±2.14	0.56±1.39	0.52±1.59	<0.001	1.54±1.82	1.29±1.49	1.17±1.51	<0.001

One-way ANOVA was used for continuous variables; Mean ± Standard Deviation was used for continuous variables.
HPHS, Harbin People health Study; HDNNCDS, the Harbin Cohort Study on Diet, Nutrition and Chronic Noncommunicable Disease.
